# Automated Detection of COVID-19 Cases on Radiographs using Shape-Dependent Fibonacci-p Patterns

**DOI:** 10.1109/JBHI.2021.3069798

**Published:** 2021-03-31

**Authors:** Karen Panetta, Foram Sanghavi, Sos Agaian, Neel Madan

**Affiliations:** Department of Electrical and Computer EngineeringTufts University 1810 Medford MA 02155 USA; Department of Computer ScienceCity University of New York 2009 New York NY 100161 USA; Department of RadiologyTufts Medical Center 1867 Boston MA 02111 USA

**Keywords:** COVID-19 detection, Fibonacci -p patterns, X-ray images, artificial intelligence, biomedical imaging, machine learning

## Abstract

The coronavirus (COVID-19) pandemic has been adversely affecting people's health globally. To diminish the effect of this widespread pandemic, it is essential to detect COVID-19 cases as quickly as possible. Chest radiographs are less expensive and are a widely available imaging modality for detecting chest pathology compared with CT images. They play a vital role in early prediction and developing treatment plans for suspected or confirmed COVID-19 chest infection patients. In this paper, a novel shape-dependent Fibonacci-p patterns-based feature descriptor using a machine learning approach is proposed. Computer simulations show that the presented system (1) increases the effectiveness of differentiating COVID-19, viral pneumonia, and normal conditions, (2) is effective on small datasets, and (3) has faster inference time compared to deep learning methods with comparable performance. Computer simulations are performed on two publicly available datasets; (a) the Kaggle dataset, and (b) the COVIDGR dataset. To assess the performance of the presented system, various evaluation parameters, such as accuracy, recall, specificity, precision, and f1-score are used. Nearly 100% differentiation between normal and COVID-19 radiographs is observed for the three-class classification scheme using the lung area-specific Kaggle radiographs. While Recall of 72.65 ± 6.83 and specificity of 77.72 ± 8.06 is observed for the COVIDGR dataset.

## I. Introduction

On march 11, 2020, the World Health Organization (WHO) declared coronavirus (COVID-19) as an pandemic due to its far-reaching seriousness throughout the world [1], [2]. As of July 27, 2020, over 16,000,000 cases and more than 600,000 deaths were recorded worldwide, with more than 250,000 cases and 5,400 deaths filed in the last 24 hours [3]. In the United States, the Center for Disease Control and Prevention (CDC) has recorded around 4,000,000 cases and more than 100,000 deaths due to coronavirus as of July 27, 2020 [4]. A real-time reverse transcriptase-polymerase chain reaction (RT-PCR) test is currently employed to detect COVID-19 cases. However, the test faces a critical problem of detecting false negatives and false positives, achieving sensitivity as low as nearly 60-70% [5]–[7]. Additionally, there is still a shortage in the availability of test kits worldwide. Moreover, the test process is labor-intensive and time-consuming and takes a long time to produce reports [8], [9]. Therefore, it generates a need for using other diagnostic approaches such as clinical investigation, epidemiological history, pathogenic analysis, computed tomography (CT), or x-ray imaging for detecting COVID-19 more quickly and effectively.

Severe COVID-19 infections exhibit similar clinical characteristics to bronchopneumonia, such as fever, cough, and dyspnea [10]–[13]. Therefore, using just the clinical investigation may not be sufficient for COVID-19 detection. Radiology imaging, such as CT or chest x-ray, is another primary tool that can be used for diagnosing COVID-19. Bilateral, multi-focal, ground-glass opacities with limited or posterior dispersal are some of the features that the majority of the COVID-19 radiology images exhibit [12], [14]–[18]. In recent studies, CT imaging has been widely used to study and detect COVID-19 cases [16], [19]. However, besides exposing the patient to a higher dosage of radiation, CT imaging is also more expensive [19].

On the contrary, x-ray imaging is cheaper and more widely available in most hospitals, making it the first-line radiologists' tool to detect COVID-19 cases [11], [19]. However, differentiating COVID-19 from other lung infections such as viral pneumonia can be very difficult for the radiologist. This lack of specificity could result in a delay of treatment and pose a danger to the patient as well as the health care providers [20]–[23]. Thus, an automated computerized system for more accurate and effective detection of COVID-19 from viral pneumonia and normal condition chest radiographs would be more invaluable.

**Fig. 1.**
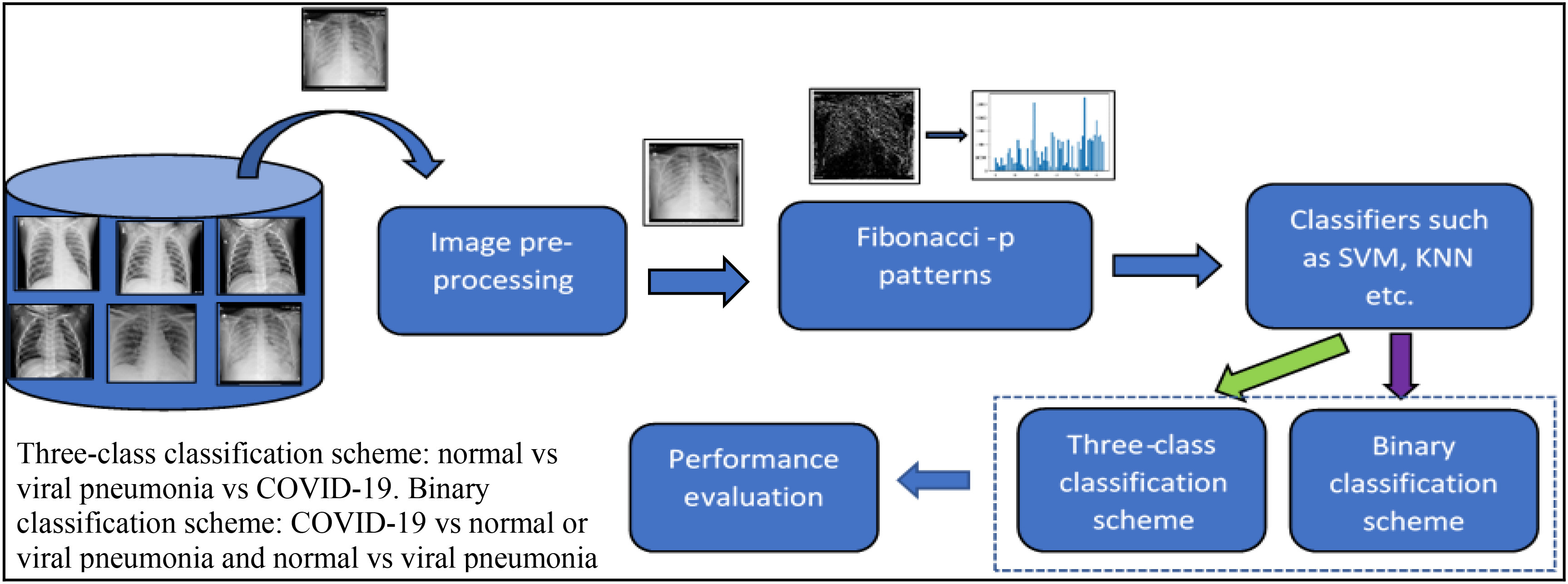
Proposed AI-based Fibonacci -p patterns-based classification system. From the directory of images, an input image is read, which is normalized in the image pre-processing step. Fibonacci image is generated using the shape-dependent Fibonacci -p pattern extractor, from which histogram is extracted and send to the classifier for training and testing purposes. Depending on the classification scheme chosen, namely, binary or three-class, classification is performed. Performance evaluation is performed on the generated confusion matrix.

Several deep learning (DL) architectures have been recently proposed to increase the accuracy in COVID-19 detection from viral pneumonia and normal radiographs. However, these methods are sophisticated and require higher computation time and resources, specialized hardware such as GPUs to train the models. DL models usually require a large training data to obtain a stable model, and given the nature of the pandemic, it is difficult to get an extensive database. Comparatively, machine learning models are simple, easy to train and deploy, and are fast. Moreover, machine learning models do not require large datasets to obtain stabilized models. Furthermore, the presence of ground-glass opacities is one of the important features seen in COVID-19 radiographs; thus, extracting textual information would help get an accurate diagnosis. Therefore, in this paper, an Artificial Intelligence (AI) based approach using shape-dependent Fibonacci -p patterns and machine learning models is proposed to effectively capture the radiographs' textural information and accurately diagnose COVID-19 (Fig. 1).

The following are the contribution of this paper:

a) A novel shape-dependent Fibonacci -p patterns-based feature descriptor to extract the underlying distinctive textural patterns, which is computationally inexpensive, tolerant to illumination changes and noise.
b) A new tool for automated detection and classification with higher accuracy that separates the COVID-19 cases from non-COVID-19 cases by using a small dataset of chest radiographs.
c) Result evaluation on the full radiographs and lung area-specific radiographs of the Kaggle dataset and the lung area-specific radiographs of the COVIDGR dataset, using evaluation metrics such as classification accuracy, precision, recall, specificity, F1 score, and confusion matrix.

The rest of the paper is organized as follows: Section II describes the current work related to COVID-19 detection with their advantages and disadvantages; Section III describes the proposed feature extractor method; Section IV describes the database used, classification models employed, and the evaluation parameters used, along with the results obtained after computer simulation; Section V concludes the paper with future work.

## II. Related Work

Presently, several DL architectures using convolutional neural network namely, COVID-Net [24], DarkNet [25], CovidX-net [26], CheXnet [27], COVID-SDnet [52] and pre-trained CNNs [2], [9], [28]–[30] have been implemented to detect COVID-19 from viral pneumonia and normal chest radiographs. The performance of these architectures on their corresponding datasets is mentioned in Table I. Even though the aforementioned methods have achieved good detection accuracies, there is still room for improvement for increasing the effectiveness in identifying COVID-19 from normal and viral pneumonia. These mentioned methods being deep-learning models usually require several hours of training time and computational hardware such as GPU's. They are usually data-hungry and complex. A machine learning approach is proposed here to overcome these shortcomings, which are lightweight models making them easier to deploy, requiring less training time, and would not require specialized hardware. Textural information plays a critical role in analyzing chest radiographs [31]. Thus, in this paper, a novel shape-dependent Fibonacci -p patterns-based texture descriptor using machine learning classification models is proposed to distinguish COVID-19, viral pneumonia, and normal chest radiographs. Additionally, a comparative analysis of the proposed method with the existing DL models for classification schemes COVID-19 vs normal and normal vs. viral pneumonia vs COVID-19 for the full radiograph Kaggle dataset is also performed. Furthermore, the proposed method's performance on lung area-specific radiographs for the Kaggle and COVIDGR dataset is also evaluated in this paper.

**TABLE I:** Literature review for the chest x-ray COVID-19 detection

Author	Dataset	Method	Classification scheme	Evaluation Results
Linda Wang *et al.* [24]	COVIDX dataset: 16756 chest x-ray images from open repositories	COVID-Net	Normal vs Non-COVID vs COVID-19	Accuracy=93.3%
Hafeez Abdul *et al.* [30]		Pre-trained ResNet50 model		Accuracy=90%
Ozturk *et al.* [25]	142 COVID-19, 500 normal, and 500 viral pneumonia images	DarkNet Model	COVID-19 vs non- finding	Accuracy = 98.08%
			COVID-19 vs non-finding vs viral pneumonia	Accuracy= 87.02%
Hemdan *et al.* [27]	25 Normal and COVID-19 images	CovidX-Net	Normal vs COVID-19	Accuracy=90% F1-score = 0.89 and 0.91 for normal and COVID-19 respectively using DenseNet201
Narin *et al.* [28]	50 COVID-19 images from GitHub dataset and 50 normal from Kaggle dataset	Pre-trained ResNet50	Normal vs Viral Pneumonia vs COVID-19	Accuracy= 97% using Inception V3 model and 87% for Inception-images ResNet V2 model
Ioannis *et al.* [29]	Dataste1: 224 COVID-19, 700 bacterial pneumonia, and 504 normal x-ray images, Dataste2: 224 COVID-19, 712 bacterial and viral pneumonia, and 504 normal x-ray images	Different fine-tuned DL models	Normal vs. COVID-19 vs. Viral Pneumonia	Accuracy=96.7%, Sensitivity=98.66%, Specificity=96.46%
Asif *et al.* [9]	864 COVID-19, 1341 normal, and 1345 viral pneumonia x- ray images	Pre-trained Inception V3 model	Normal vs. COVID-19 vs. Viral Pneumonia	Accuracy=96%
**Chowdhury *et al.* [2]**	**Kaggle Dataset: 219 COVID-19, 1341 Normal, and 1345 Viral Pneumonia**	**Pre-trained CNN models**	**Normal vs COVID-19**	**Accuracy= 98.3%**
			**Normal vs COVID-19 vs. Viral Pneumonia**	**Accuracy=98.3%**
**Bassi *et al.* [27]**		**CheXNet**	**Normal vs COVID-19 vs Viral Pneumonia**	**Accuracy=97.8%**
**Tabik et. al [52]**	COVIDGR dataset: 852 images with 426 images non-COVID-19 and COVID-19	**COVID-SDnet**	**Non-COVID vs COVID**	**Accuracy=**$76.18\pm 2.70$

## III. Shape-Dependent Fibonacci-p Patterns

**Local Binary Patterns (LBP)** is a texture descriptor that utilizes the center pixel's information and its respective neighboring pixels to encode the structural and statistical texture information present in an image [32]. Herein, an image is first divided into overlapping windows of neighborhood mxn and for each of these windows, the center pixel and its surrounding n neighboring pixels are compared. Suppose the latter is greater than or equal to the center pixel. In that case, it is binarized as ‘one’ or else as ‘zero.’ A binary pattern obtained by combining these binary numbers is then converted into a decimal value by assigning the appropriate decimal weights and summing them together, thus encoding the textural information present in the window [33], [34] and subsequently obtaining an LBP image. The following are the advantages of classical LBP; (a) it is simple, fast, and easy, and (b) is insensitive to illumination changes. However, it suffers the disadvantages of intolerance to noise and computational expensiveness due to longer feature vector dimensionality. To overcome these shortcomings, a Fibonacci -p patterns-based descriptor is proposed.

**Fibonacci-p patterns** are textural feature descriptors that work very similar to LBP, i.e., they also encode the textural pattern information surrounding every pixel present in an image by assigning appropriate Fibonacci weights to them [35]. However, the difference between LBP and Fibonacci -p patterns is that in the latter, a set threshold value is used for binarizing the mxn neighborhood. If the difference between the center pixel and its respective neighboring pixels is greater than or equal to the set threshold value, the neighboring pixel is binarized as ‘one,’ or else is binarized as ‘zero’. To generate the decimal value, Fibonacci weights are assigned to the obtained binary pattern and summed together. Thus, generating a Fibonacci image. The following is the mathematical formula used for computing Fibonacci -p patterns [36]–[38]:

\begin{align*}
Fib_{r, k}=&\sum\limits_{i=0}^{k-1}s_{i}\ast f_{p}(i) \tag{1}\\
s_{i}=&\begin{cases}
1, & \qquad {\rm{if}}\;(b_{i}- b_{c})\geq T,\\
0, & \qquad {\rm{otherwise}}
\end{cases}\tag{2}\\
f_{p}(i)=&\begin{cases}
0, & i < 0\\
1, & i=0\\
f_{p}(i-1)+f_{p}(i-p-1), & i > 0
\end{cases} \tag{3}
\end{align*}

Where, $k$ = number of neighbors and $r$ = radius, p= pattern value, $f_{p}$ = Fibonacci weights and $T$ = set threshold. Table II shows the Fibonacci weights computed using (3) depending on the p-value.

Thresholding plays an integral role while computing Fibonacci -p patterns as it helps in eliminating noise while extracting the textural patterns from the images. The threshold value determines the extent of information be incorporated in the patterns, i.e., the higher the threshold value, the less the information becomes incorporated and vice versa [39]. The Fibonacci-p patterns serve the following advantages: (a) when $\mathrm{p}=0$, the weights obtained are similar to LBP, thus, behaving as an LBP operator, which is another textural descriptor, (b) it is insensitive to the illumination changes, (c) it is insensitive to noise because of the utilization of threshold value in the binarization process, (d) it is computationally inexpensiveness due to reduction in feature vector dimensionality for $\mathrm{p} > 0$ (Table II), and (e) it has the flexibility to add more information due to lower feature vector for $\mathrm{p} > 0$ [35].

**Fig. 2.**
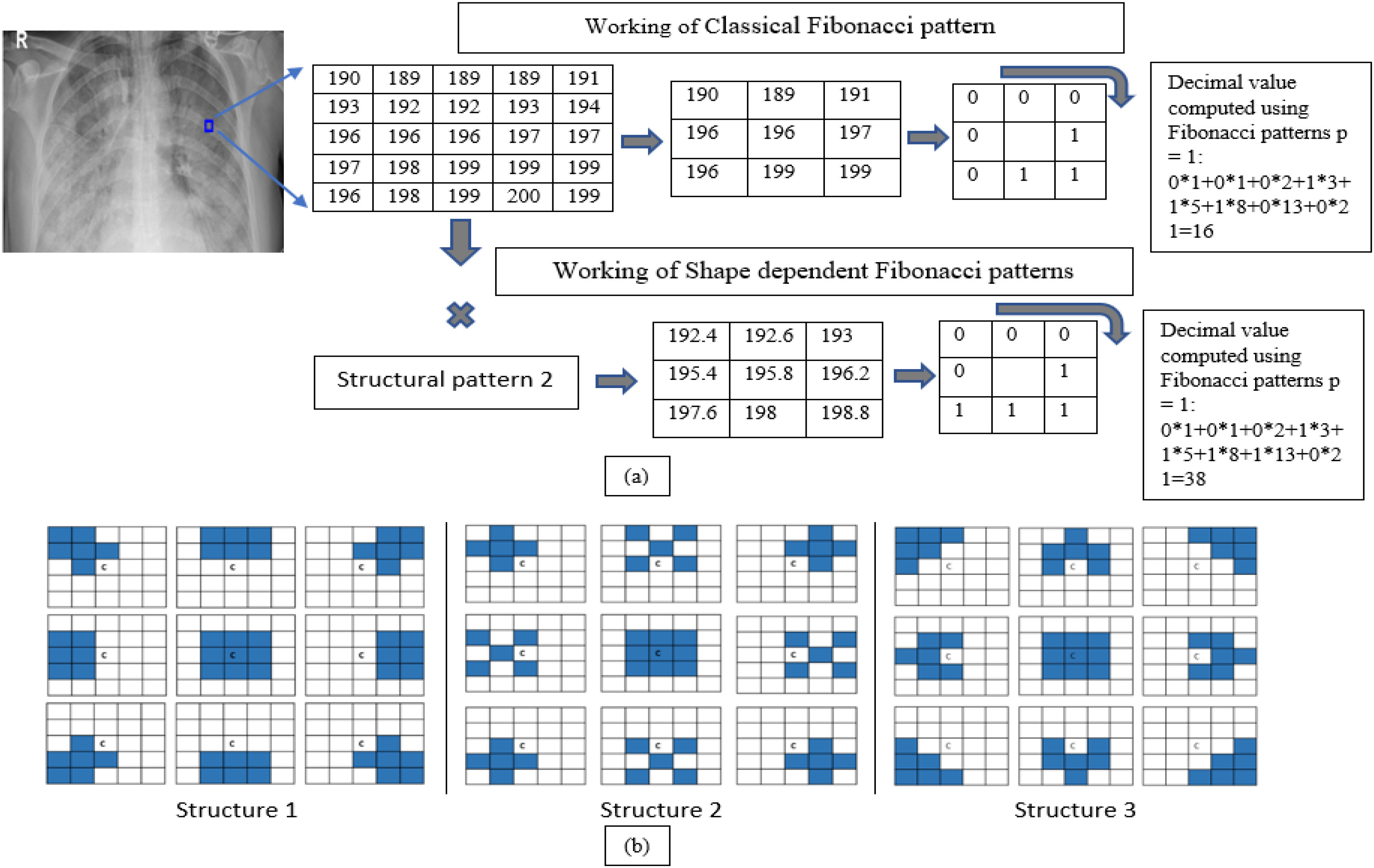
Schematic representation of working of Fibonacci -p patterns and shape-dependent Fibonacci -p patterns using $\mathrm{T}=1$, and 8 neighbors. The highlighted areas have the value ‘one,’ and the rest are ‘zeros’.

However, for a window size larger than $3\times 3$, not all pixels in the given window gets included while computing the Fibonacci-p patterns. For example, for a $5\times 5$ window using k neighbors, only k pixels get encoded in the pattern information, missing out information present in the remaining pixels, which could be important. Moreover, the classical Fibonacci fails to encode patterns having various shapes except for the circular pattern. To solve this problem, Shape-dependent Fibonacci -p patterns-based feature descriptor is proposed.

**Shape-dependent Fibonacci -p patterns** use the different shapes given in the structural pattern (Fig. 2(b)) to encrypt the textural information present in an image. To compute shape-dependent Fibonacci -p patterns, the image is first divided into windows of mxn neighborhoods. From each window, information is extracted as per the highlighted area present in the structural pattern's nine shapes. The arithmetic mean is computed for each shape information and arranged similarly to the structural pattern. Fibonacci -p patterns are then computed using equations (1), (2), and (3). This ensures that most of the information present in the mxn neighborhood is taken into account. Fig. 2(a) illustrates the working of classical Fibonacci -p patterns and the Shape-dependent Fibonacci -p pattern $5\times 5$ neighborhood, and Fig. 2(b) shows the three different structural patterns that are experimented with within this paper. Only the 8 neighbors that lie on the circle's circumference of radius $\mathrm{r}=2$ get encoded in the classical Fibonacci case. However, it is unknown if the point getting encoded is a random noise point or a textural pattern point. To mitigate this, the Shape-dependent Fibonacci -p patterns encode the localized area information instead. This is because the average values of the texture data extracted using the structural pattern's shape information is used for encoding.

**TABLE II:** Fibonacci weights computed for different p values [36]

	$i$									
$p$	0	1	2	3	4	5	6	7	8	…
0	1	2	4	8	16	32	64	128	256	…
1	1	1	2	3	5	8	13	21	34	…
2	1	1	1	2	3	4	6	9	13	…
3	1	1	1	1	2	3	4	5	7	…
4	1	1	1	1	1	2	3	4	5	…
:	:	:	:	:	:	:	:	:	:	…
∞	1	1	1	1	1	1	1	1	1	1

Since the shape of the disease pattern to be encoded can be arbitrary, using the points lying circularly may not be enough to highlight all the edges, curves, and edge ends. Therefore, the shape-dependent-p patterns will be more beneficial as the information is extracted using different shapes. Furthermore, in contrast to classical Fibonacci, the center pixel information also gets encoded here.

**Fig. 3.**
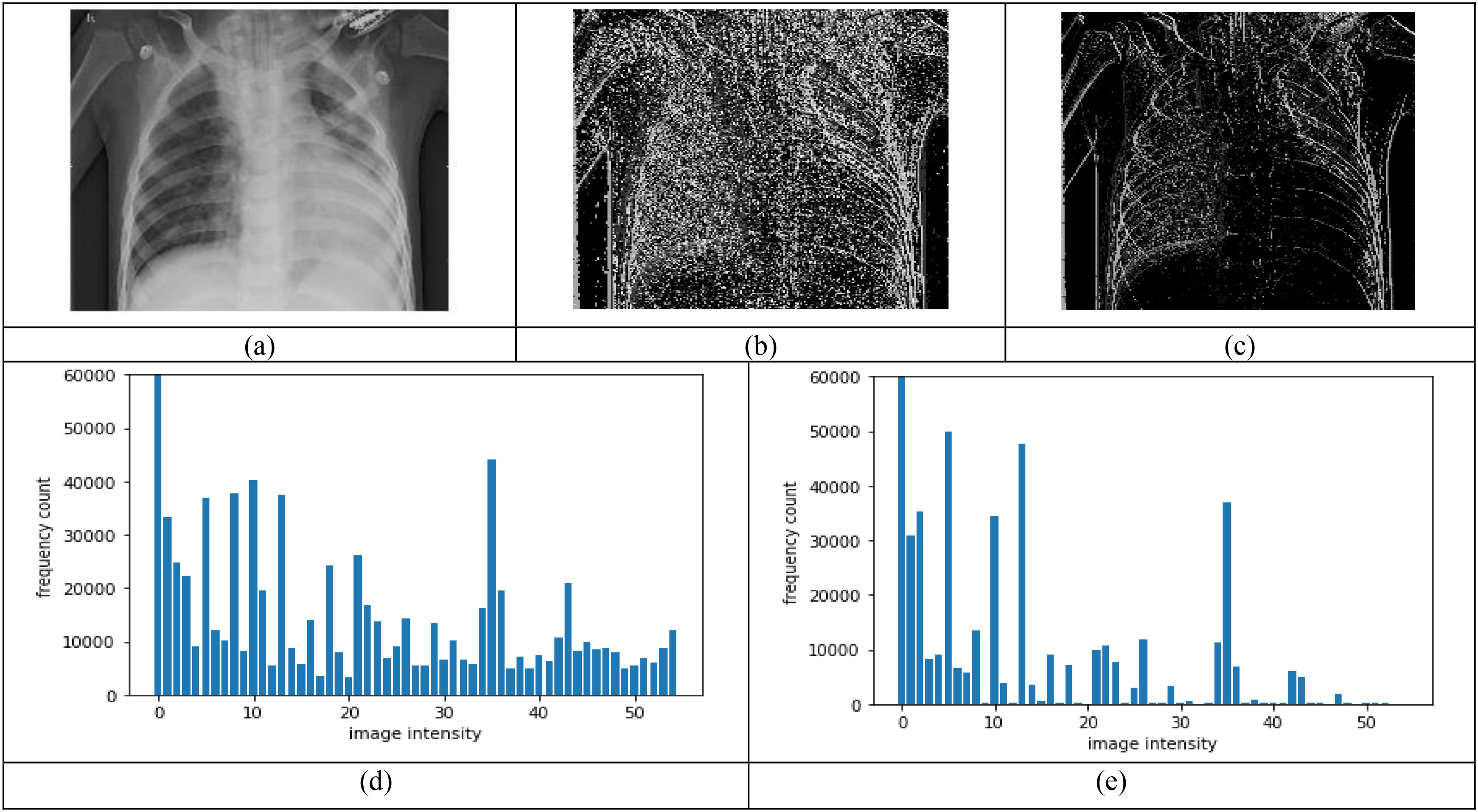
Comparison between classical Fibonacci and shape-dependent Fibonacci p patterns, using a set window size of ${\text{5}}\times {\text{5}},\ \mathrm{T} = {\text{2}}$, and $\mathrm{p}= {\text{1}}$, (a) Original image, (b) Classical Fibonacci image, (c) Shape-dependent Fibonacci p image using structural pattern 2, (d-e) Histograms obtained from the images in (b) and (c) respectively.

The main advantage of shape-dependent Fibonacci -p patterns is the encoding of the textural patterns aligned in different directions and shapes in the image all in one operation. In addition, the arithmetic means computation performed behaves like mean filtering, inherently eliminating the noise present in the image. Another advantage of Shape-dependent Fibonacci -p patterns is that they can detect different textures and discontinuities such as spots, flat areas, edges and edge ends, and curves. Thus, the Shape-dependent Fibonacci patterns concept's key benefit is capturing more textural information than the classical Fibonacci case, making it a more data-adaptive and context-aware image descriptor. Fig. 3 shows the performance between the classical Fibonacci operator and the shape-dependent Fibonacci operator on COVID-19 radiographs.

**Fig. 4:**
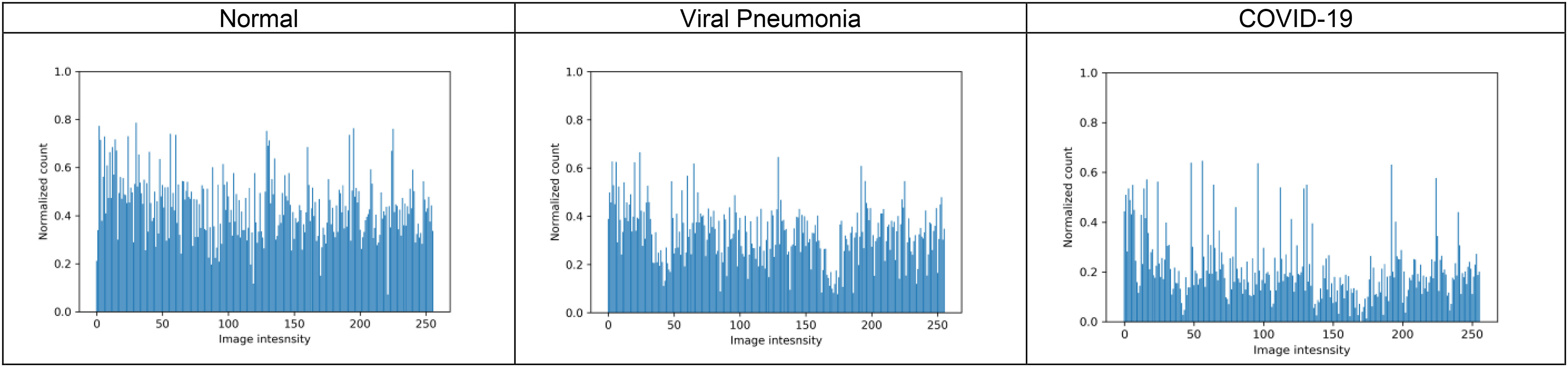
Illustration of histograms of normal, viral pneumonia and COVID-19 radiographs from full radiograph kaggle dataset for pattern value $\mathrm{p}={\text{0}}$, window size ${\text{5}}\times {\text{5}}$ and $\mathrm{T}={\text{1}}$.

**Fig. 5.**
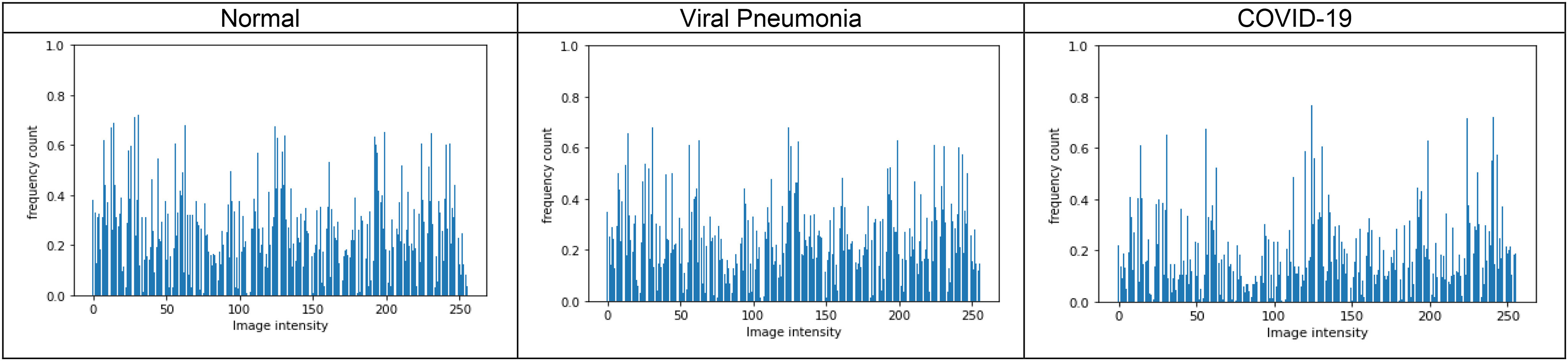
Illustration of histograms of normal, viral pneumonia and COVID-19 radiographs from lung area-specific kaggle dataset, for pattern value p = 0, window size ${\text{5}}\times {\text{5}}$ and $\mathrm{T}={\text{0}}$.

It can be observed that for a set window size of $5\times 5$, threshold of 2, and $\mathrm{p}=1$, the Shape-dependent Fibonacci operator has less noise encoded compared to the classical case, which reflects on the histogram feature extracted from the Fibonacci images. The histogram feature obtained from the shape-dependent Fibonacci images of all three classes is then scaled between 0 and 1 and sent to the classifier for training and testing purposes. Fig. 4 illustrates the histograms computed from the full normal, viral pneumonia and COVID-19 radiographs present in the Kaggle dataset. Fig. 5 illustrates the histograms computed from the normal, viral pneumonia and COVID-19 radiographs from the lung area-specific radiograph Kaggle dataset. The histograms shown is computed over 20 images and averaged for each class.

## IV. Computer Simulations and Experimental Results

### A. Dataset Description

**Fig. 6.**
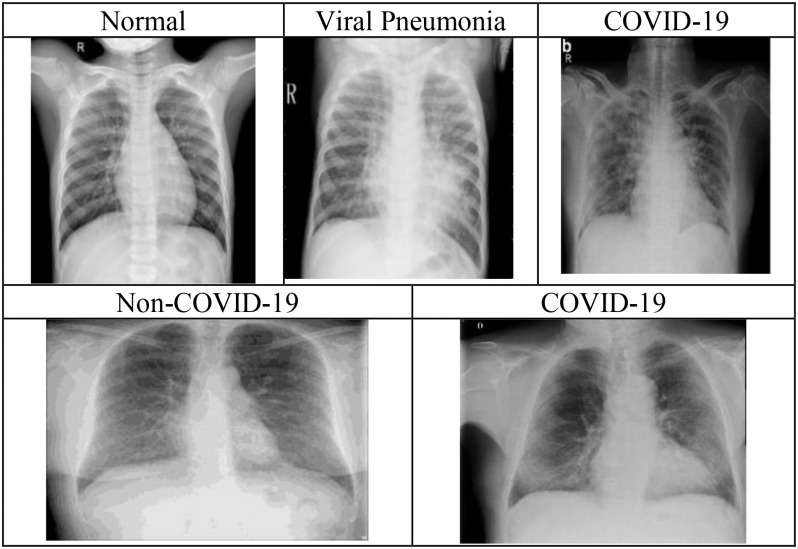
Illustration of chest radiographs present in the Kaggle dataset (top row) and COVIDGR dataset (bottom row).

The first dataset (Kaggle dataset) comprises of 219 COVID-19, 1345 viral pneumonia, and 1341 normal chest radiographs, obtained from the publicly available Kaggle website [2]. The authors collected the COVID-19 chest radiographs in [2] from different sources [40], [41], and the different articles published related to coronavirus. Similarly, viral pneumonia and normal chest radiographs used in the dataset were collected from the publicly available dataset on the Kaggle website [42]. Each radiograph in the database is of the size $1024^{\ast}1024$. The second dataset (COVIDGR dataset) comprises 852 chest radiographs, with both positive (Covid-19) and negative (Non-Covid-19) class containing 426 chest radiographs [52]. All the images in this dataset were acquired from the same X-ray machine, and the chest radiographs were labeled as COVID-19 only when both the RT-PCR test and the radiologist confirmed the results within a day [52]. Image normalization is performed here to ensure that all the images lie in the same contrast range so that the classification system's effectiveness is not affected. Fig. 6 illustrates the radiographs present in Kaggle Dataset and COVIDGR dataset.

### B. Feature Extraction and Training

The images are read sequentially from the image directories present in the dataset on which normalization is performed. The Shape-dependent Fibonacci features are extracted from these normalized images, and the extracted feature matrix with its corresponding labels is randomly shuffled and split into training, testing, and validation sets. Six different machine learning classifiers, namely SVM [43], KNN [44], Random Forest [45], AdaBoost [46], Gradient Tree Boosting [47], and Decision Tree [48], are used for training purposes. For the above-mentioned classifiers, automated hyper-parameter tuning with appropriate cross-validations is performed, and the classifier model giving the best result is automatically selected. For the Kaggle dataset, the feature matrix with its corresponding labels is randomly shuffled and split into 70% training and30% testing sets, and 10% of the training data as a validation set. Hyper-parameter tuning is performed using 10 cross-fold validation. For the COVIDGR dataset, the feature matrix with its corresponding labels is randomly shuffled and split into 90% training, and 10% testing sets 10% of the training data as validation. The Hyper-parameter tuning is performed using 5 cross-fold validation.

### C. Performance Evaluation

The best-selected classifier model's performance is evaluated using different parameters, namely, accuracy, sensitivity (recall), specificity, precision, and f1-score. The following are the formulae for computing the parameters mentioned above [49], [50]:

\begin{align*}
Accuracy=&\frac{Tp+Tn}{Tp+Tn+Fp+Fn}\ast 100\tag{4}\\
Recall=&\frac{Tp}{Tp+Fn}\ast 100\tag{5}\\
Specificity=&\frac{Tn}{Tn+ Fp}\ast 100\tag{6}\\
Precision=&\frac{Tp}{Tp+Fp}\ast 100\tag{7}\\
F1-score=&\frac{2\ast precision\ast recall}{precision+recall}\tag{8}
\end{align*}

where, $Tp$, and $Tn$ are the number of classes correctly classified as positive and negative classes respectively, and $Fp$, and $Fn$ are the number of images falsely classified as positive and negative classes, respectively.

### D. Results

For the Kaggle dataset, four different classification schemes are implemented in this paper, namely, COVID-19 vs viral pneumonia, COVID-19 vs normal, normal vs viral pneumonia, and normal vs viral pneumonia vs COVID-19 chest radiographs. Since the dataset used here is imbalanced, using accuracy as the only tool to measure the effectiveness of the feature extractor would not be enough. Furthermore, how truly the model can distinguish COVID-19, viral pneumonia, and normal chest radiographs from each other is also a critical factor to be measured. Thus, using parameters like recall, specificity, precision, and f1-score is of more significance. To select the optimal pattern (p) and threshold value(T) for the above-mentioned classification schemes, their values are varied from 0–3, and values giving the best precision-recall performance are chosen. Similarly, the optimal structural pattern is selected for evaluating the precision-recall performance of all three proposed structural patterns.

**Fig. 7.**
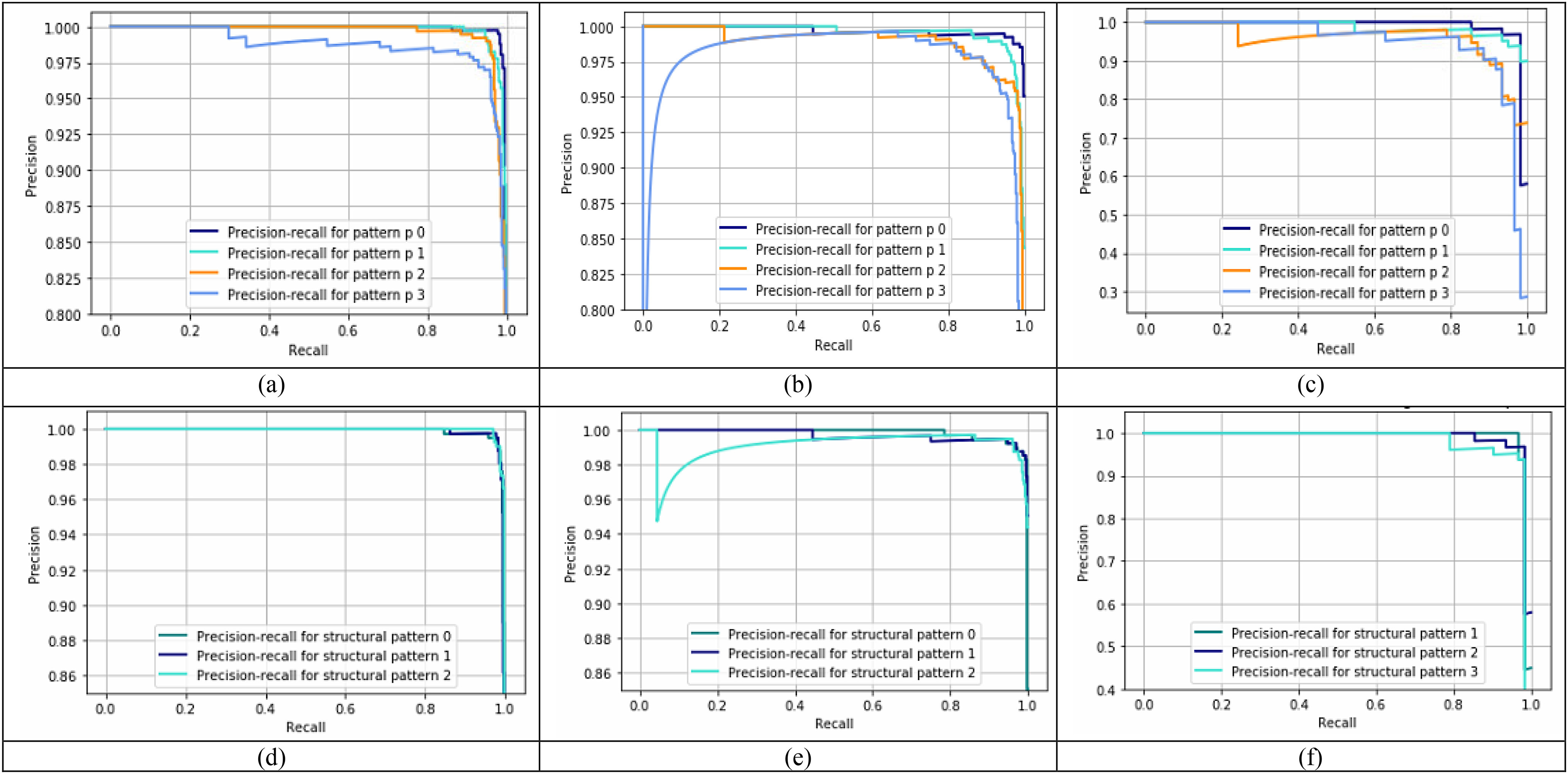
Precision-recall curves using set window size of ${\text{5}}\times {\text{5}}$ and $\mathrm{T}= {\text{1}}$ for the three-class classification scheme on the full radiograph kaggle dataset. (a), (b), and (c) show the precision-recall performance of different pattern (p) values on normal, viral pneumonia and COVID-19 images respectively using structural pattern 2, and (d), (e), and (f) show the precision-recall performance of different structural patterns for normal, viral pneumonia and COVID-19 images respectively using $\mathrm{p}= {\text{0}}$.

Fig. 7 illustrates the precision-recall curves plotted to assess the performance of different p values and structural patterns for the three-class classification scheme, i.e., normal vs viral pneumonia vs COVID-19. Fig. 7(a), (b), and (c), show the precision-recall curves for different p values for the normal, viral pneumonia, and COVID-19 cases, respectively. It can be observed that $\mathrm{p}=0$ gives the best performance for all three cases. To generate these curves, the window size and threshold value was set to $5\times 5$ and 1, and the structural pattern 2 is used. Likewise, Fig. 7(d), (e), and (f), show the precision-recall curves of different structural patterns on normal, viral pneumonia, and COVID-19 cases, respectively.

**Fig. 8.**
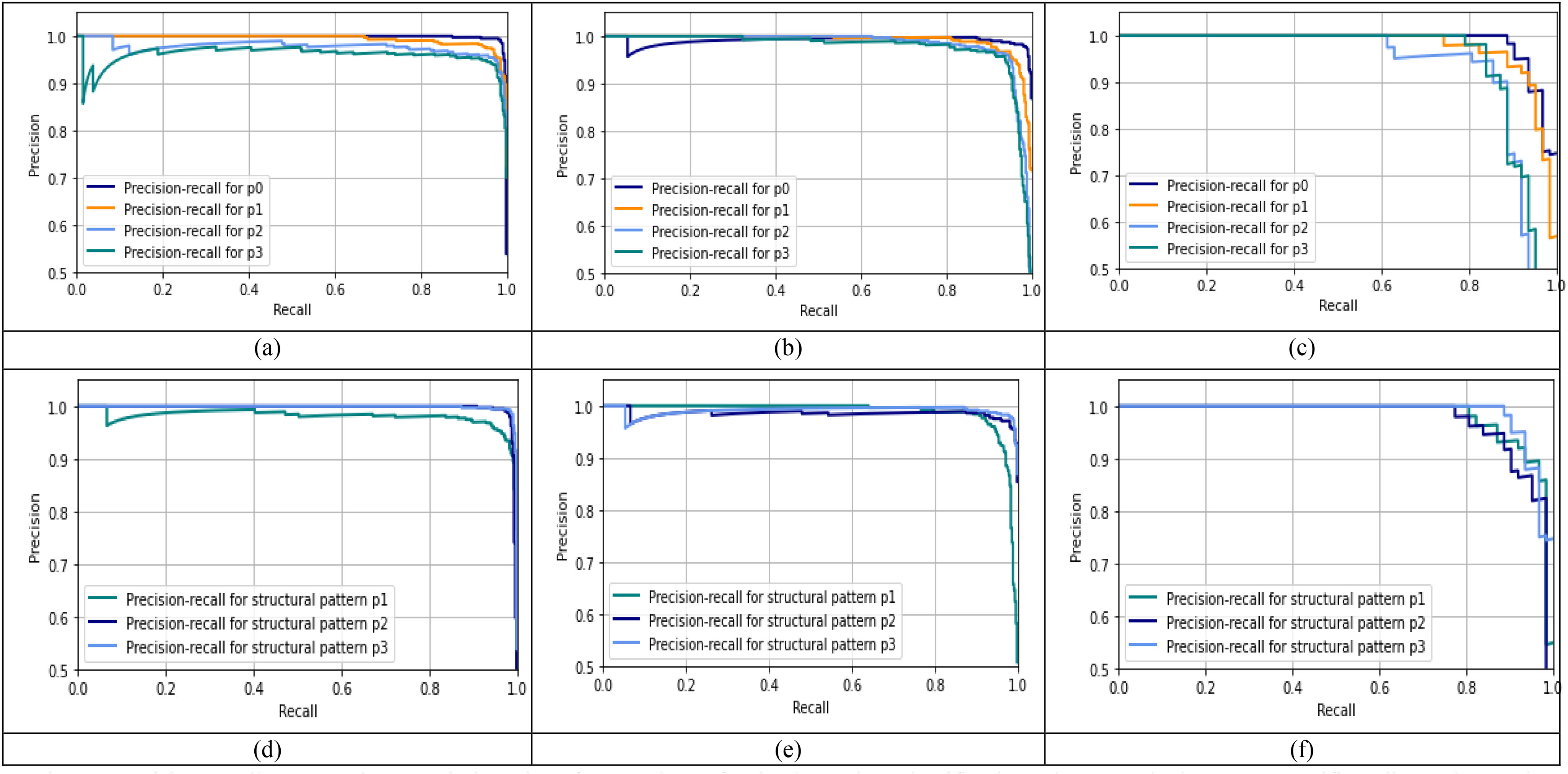
Precision-recall curves using set window size of ${\text{5}}\times {\text{5}}$ and $\mathrm{T}={\text{0}}$ for the three-class classification scheme on the lung area-specific radiograph Kaggle dataset. (a), (b), and (c) show the precision-recall performance of different pattern (p) values on normal, viral pneumonia and COVID-19 images respectively using structural pattern 3, and (d), (e), and (f) show the precision-recall performance of different structural patterns for normal, viral pneumonia and COVID-19 images respectively using $\mathrm{p}={\text{0}}$.

Similar performance is observed for all three structural patterns for normal and viral pneumonia images, but structural pattern 2 yields better performance for COVID-19 images. To generate these curves, the window size and threshold value were set to $5\times 5$ and 1, and $\mathrm{p}=0$ was used. The optimal parameters for the other classification schemes were obtained similarly. Computer simulations show that for the classification schemes, COVID-19 vs viral pneumonia and normal vs viral pneumonia, $\mathrm{p}=0,\ \mathrm{T}=2$, and structure pattern 2 gives the best results, whereas, for the classification scheme, COVID-19 vs normal, both $\mathrm{p}=2,\ \mathrm{T}=1$ and $\mathrm{p}=1,\ \mathrm{T}=2$, and structural pattern 2 yields the best outcome. Table III(A) illustrates the performance of the proposed feature extractor and the DL based methods utilized for the Kaggle dataset for the classification schemes COVID-19 vs normal and normal vs viral pneumonia vs COVID-19.

It can be observed that the proposed method achieves better performance by nearly 5–7% for detecting COVID-19 images (recall) for the COVID-19 vs regular classification scheme. A high sensitivity, thereby correctly identifying most of the COVID-19 images is preferable in the current pandemic climate. Similarly, for the three-class classification scheme, the proposed method shows improved performance of 1–5% and 1–4% in recall and specificity, respectively, as compared to methods used by Chowdhury *et al.* [2] and Bassi *et al.* [27]. Likewise, compared to the classical Fibonacci -p pattern, the proposed Shape-dependent Fibonacci -p pattern yields better recall results for both the classification schemes. This is because the conventional Fibonacci -p patterns fail to incorporate the alignment and shape of the textural patterns that are required for more accurately distinguishing the three classes from each other.

**TABLE III(A):** Comparative analysis of the proposed method with DL based methods used on the full radiograph Kaggle dataset

Classification scheme	Author	Method	Accuracy	Recall	Specificity	Precision	F1-score
COVID-19 vs Normal	Chowdhury *et al.* [2]	AlexNet	97.5%	95%	100%	100%	97%
		ResNet18	96.7%	93.3%	100%	100%	96.5%
		DenseNet210	97.5%	95%	100%	100%	97.4%
		SqueezeNet	98.3%	96.7%	100%	100%	98.3%
	Our work*	**Classical Fibonacci p patterns**	**99.78%**	**98.21%**	**100%**	**100%**	**99.09%**
		**Shape-dependent Fibonacci -p patterns**	**99.78%**	**100%**	**99.76%**	**98.25%**	**99.12%**
Normal vs viral pneumonia vs COVID-19	Chowdhury *et al.* [2]	AlexNet	95.4%	93%	95.8%	100%	95.5%
		ResNet18	95%	95%	96%	100%	97.4%
		DenseNet210	96.7%	96%	96%	98.3%	97.1%
		SqueezeNet	98.3%	96.7%	99%	98%	98.3%
	Bassi *et al.* [27]	CheXNet	97.8%	97.8%	-	100%	97.8%
	Our work*	**Classical Fibonacci -p patterns**	**98.74%**	**97.73%**	**99.27%**	**98.17%**	**97.95%**
		**Shape-dependent Fibonacci -p patterns**	**98.88%**	**98.72%**	**99.35%**	**98.29%**	**98.50%**

**TABLE III(B):** Performance analysis of the proposed method on the lung area-specific radiograph kaggle dataset for normal vs COVID-19 classification

Classification scheme	Author	Method	Accuracy	Recall	Specificity	Precision	Fl-score
Normal vs COVID-19	Our work*	**Classical Fibonacci p patterns**	**99.78%**	**98.08%**	**100%**	**100%**	**99.03%**
		**Shape-dependent Fibonacci -p patterns**	**99.78%**	**100%**	**99.75%**	**98.27%**	**99.13%**
Normal vs viral pneumonia vs COVID-19	Our work*	**Classical Fibonacci -p patterns**	**97.79%**	**96.90%**	**98.52%**	**97.78%**	**97.32%**
		**Shape-dependent Fibonacci -p patterns**	**98.03%**	**96.76%**	**98.86%**	**97.20%**	**96.69%**

Table IV shows the performance of the proposed method for the classification schemes COVID-19 vs viral pneumonia, and normal vs viral pneumonia, from which high recall and specificity values can be noted. Thus, 98.44% of the time COVID-19 images will be correctly classified concerning viral pneumonia images, and 98.50% of the time, viral pneumonia images will get correctly identified from the normal images. Similarly, 99.26% of the time, viral pneumonia images will get correctly distinguished from COVID-19 images. Confusion matrices play a critical role in understanding how the classification models work on the test data. Different evaluation parameters are computed using the information obtained from it. Fig. 10 shows the normalized confusion matrices for the aforementioned classification schemes for the full radiograph Kaggle dataset, which can validate the above tables' results.

### E. Results Obtained for the Lung Area-Specific Radiograph Kaggle Dataset

**Fig. 9.**
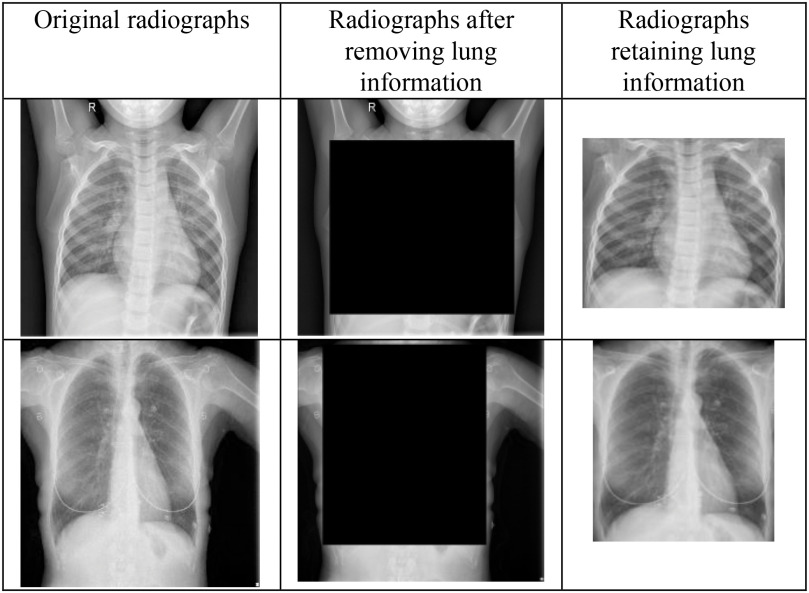
Illustration of full radiographs (left), radiographs after cropping lung information (middle), and radiographs having just the lung information (right) for the Kaggle dataset (top) and COVIDGR dataset (bottom).

Cohen *et al.* in [53] noticed that the testing protocols may be learning the dataset-specific information rather than disease-specific information on generalizing chest radiographs prediction across multiple datasets. Currently, the majority of COVID-19 detection and recognition papers have combined the COVID-19 images from the dataset in [54] with the existing non-COVID-19 datasets. In [51], the authors proposed a protocol to test whether the COVID-19 prediction model learn dataset specific or disease-specific information when used across multiple datasets. Herein, the lung information is removed by blackening the center of the chest radiographs obtained from different datasets and AlexNet is trained to see if it can identify the source of the dataset. It was observed that if both the training and testing set contained images from the same dataset, AlexNet was able to distinguish them very accurately. The solution recommended for this problem was to find dataset datasets with similar features or find a pre-processing method to delete dataset-specific information. Thus, in this paper, to delete the dataset-specific information, the chest radiographs from the Kaggle dataset and COVIDGR dataset are hand cropped to retain the lung information i.e., the disease-specific information. Hence, generating the lung area-specific radiograph Kaggle and COVIDGR dataset. The proposed feature descriptor is tested on them, and its performance is evaluated. Fig. 9 illustrates the sample images present in lung area-specific Kaggle and COVIDGR dataset. This dataset is available on Kaggle website (www.kaggle.com/dataset/ab84db1d9bab332bb7d6e2bd89a287c0b712144423f9f773e1924c62255099d4)?.

**TABLE IV:** Performance analysis of COVID vs viral pneumonia and viral pneumonia vs normal classification schemes on the full radiograph and lung area-specific radiograph Kaggle dataset

	Full radiograph Kaggle dataset		Lung area-specific radiograph Kaggle Dataset	
	COVID-19 vs Viral Pneumonia	Normal vs Viral Pneumonia	COVID-19 vs Viral Pneumonia	Normal vs Viral Pneumonia
Accuracy	99.14%	98.88%	99.13%	98.01%
Recall	**98.44%**	**98.50%**	**98.28%**	**98.52%**
Specificity	**99.26%**	**99.26%**	**99.26%**	**97.50%**
Precision	95.45%	98.53%	95.00%	97.56%
F1-score	96.92%	98.90%	96.61%	98.04%

Fig. 8 illustrates the precision-recall curves obtained for the three-class classification scheme for the lung area-specific radiograph Kaggle dataset. Fig. 8(a), (b), and (c) show the precision-recall curves for different p-values using a fixed structural pattern and Fig. 8(d), (e), and (f), show the precision-recall curves for different structural patterns using a fixed p-value; for the normal, viral pneumonia, and COVID-19 classes, respectively. To generate these curves, a fixed threshold value of 0 and a window size $5\times 5$ is used. It can be observed that for a three-class classification scheme, structural pattern 1 gives a better recall performance for the COVID-19 radiographs but has a low recall for viral pneumonia and normal class. However, structural pattern 3 yields better performance for all three classes. On comparing the precision-recall curves obtained for the lung area-specific radiograph and full radiograph Kaggle dataset, a similar performance is observed for normal and viral pneumonia class, with a slight decrement in COVID-19 class detection performance. This results in a minor decrement of 2% in the overall recall performance of the three-class classification scheme while having similar specificity performance as compared to the full radiograph Kaggle dataset, which can be seen from Table III (A) and Table III(B).

However, for the two-class classification schemes, namely COVID-19 vs normal and COVID-19 vs viral pneumonia, comparable recall-specificity performance for COVID-19 detection can be observed for both lung area-specific radiograph and full radiograph Kaggle datasets, which can be seen from Table III and IV. Whereas, for the normal vs. viral pneumonia classification scheme, a decrement of around 2% in specificity can be observed, while having similar recall performance. Computer simulations show that for the classification schemes, COVID-19 vs. viral pneumonia and normal vs. COVID-19, $\mathrm{p}=0$, structure pattern 1 and $\mathrm{T}=3$ gives the best results, whereas, for the classification scheme, normal vs. viral pneumonia, $\mathrm{p}=0,\ \mathrm{T}=0$, and structural pattern 2 yield the best outcome.

Fig. 11 shows the normalized confusion matrices for the aforementioned classification schemes for the lung area-specific radiograph Kaggle dataset, which can validate the above table's results. From the confusion matrices, nearly 100% detection between normal and COVID-19 class can be observed using disease-specific information from the radiographs in the three-class classification scheme.

**Fig. 10.**
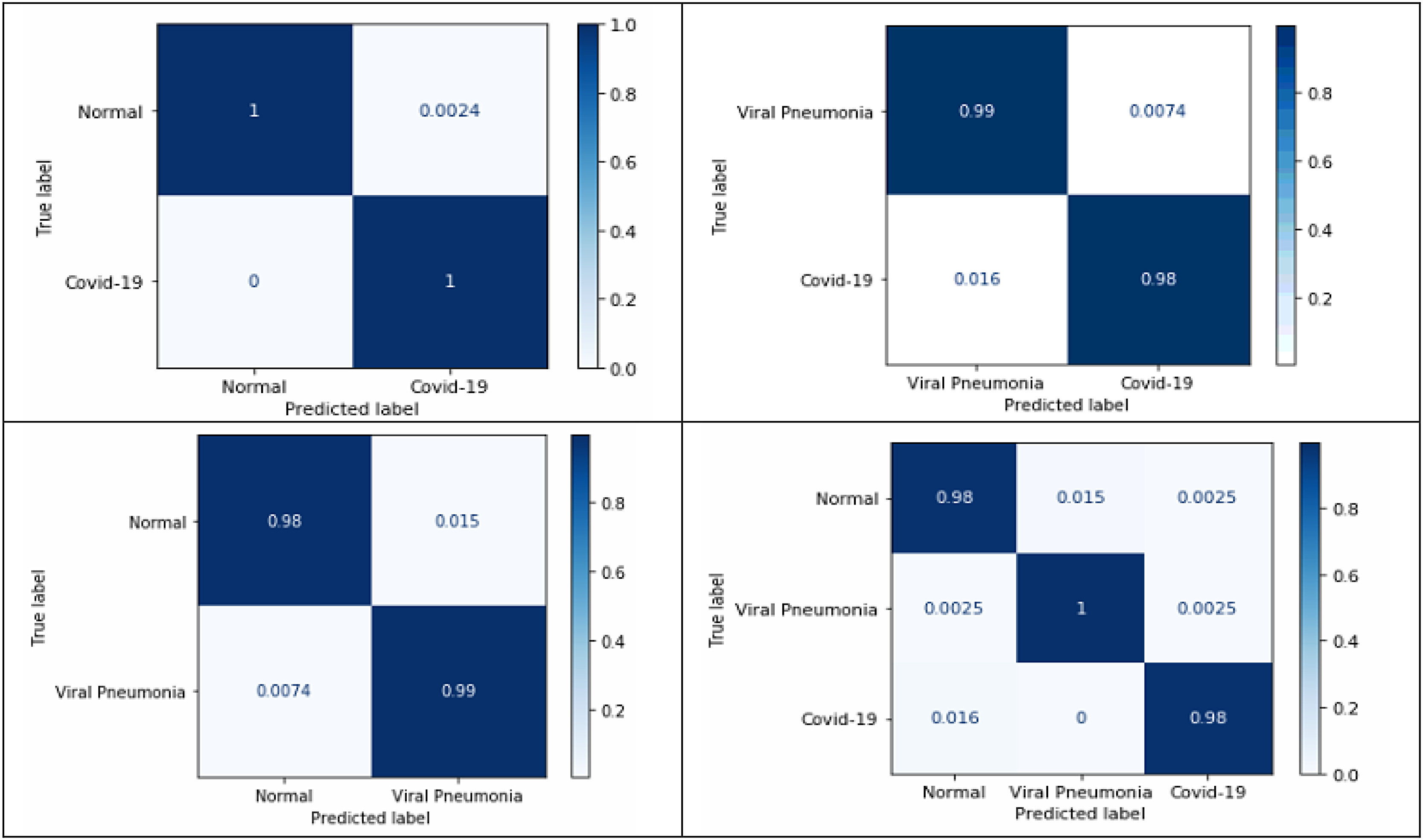
Normalized confusion matrices for the proposed four classification schemeson full radiograph Kaggle dataset.

**Fig. 11.**
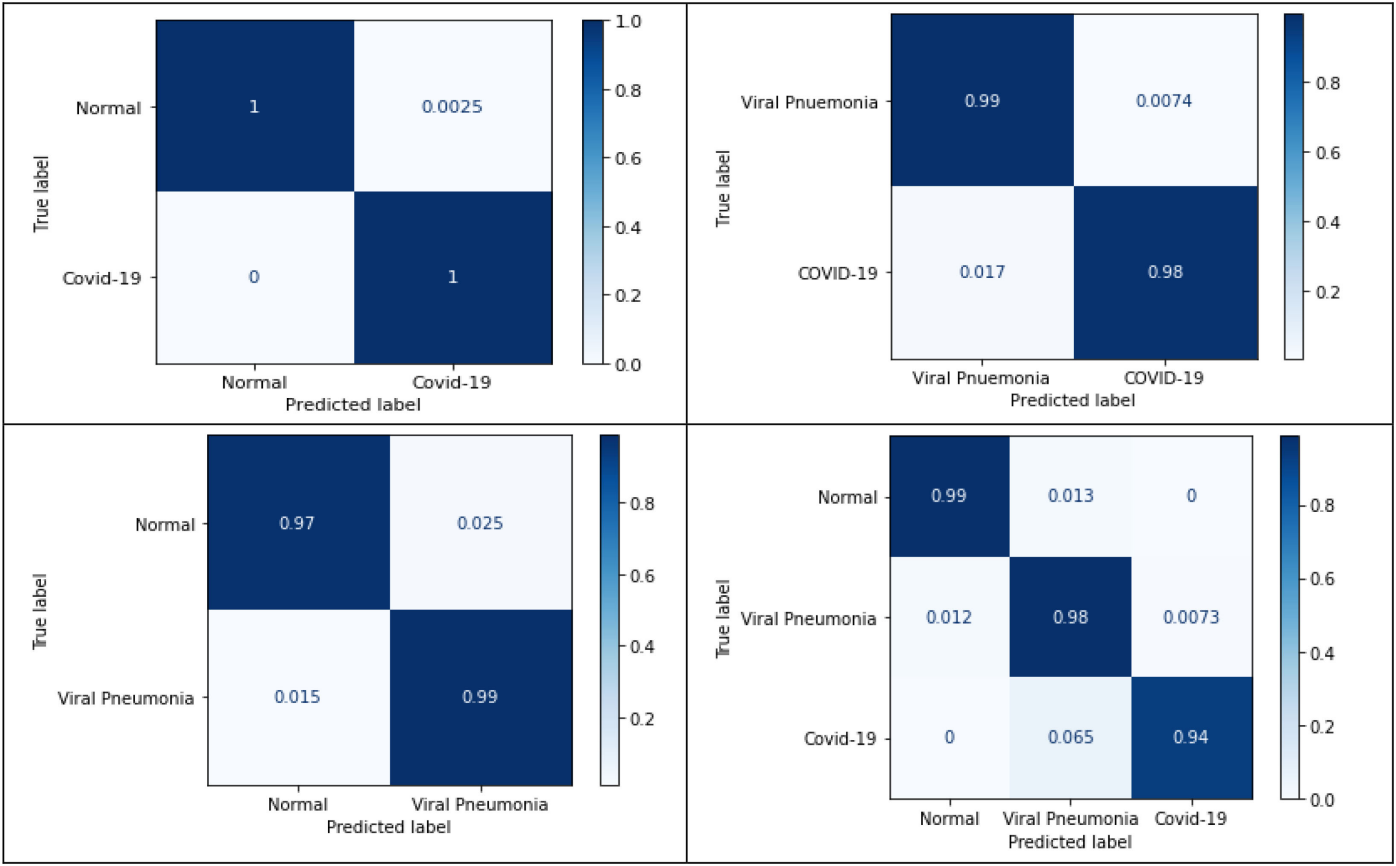
Normalized confusion matrices for the proposed four classification schemes on lung area-specifc radiograph Kaggle dataset.

### F. Performance Evaluation on COVIDGR Dataset

For the COVIDGR dataset, two-class classification scheme namely, COVID-19 vs. non-COVID-19 is proposed in this paper. The optimal parameter selection process for this dataset follows the same procedure as described for the Kaggle dataset. The proposed feature extractor's performance is evaluated using the average and standard deviation values for each of the mentioned parameters over the 5 different executions performed on the 5-cross validation. Table V illustrates the proposed feature descriptor's performance and the authors' DL methods in the article [53]. It can be observed that structural pattern 3 gives the best recall performance, but it has low specificity performance. However, structural pattern 2 yields a better-balanced recall-specificity performance. Moreover, it can be observed that the structural pattern 2 yields better recall and specificity performance than most DL methods, namely COVIDNet-CXR, COVID-CAPS, ResNet50 without segmentation, and FuCiTNet, while achieving comparable results with respect to the COVID-SDNet.

## V. Conclusion

This paper proposes a machine learning-based approach using a novel textural feature descriptor, Shape-dependent Fibonacci-p patterns for effectively distinguishing COVID-19, viral pneumonia, and normal condition chest radiographs from each other. This descriptor's key advantage is that it can encrypt textural patterns having different shapes, orientations, and discontinuities in one operation while inherently removing noise from the image. Computer simulations for the full radiograph Kaggle dataset show that the proposed method has better recall performance than the DL methods and the classical Fibonacci descriptor. Nearly 100% and 98.44% COVID-19 detection accuracy are achieved for the classification schemes COVID-19 vs normal and COVID-19 vs viral pneumonia, respectively. For the lung area-specific radiograph Kaggle dataset, similar performance was observed for COVID-19 detection for the classification schemes COVID-19 vs normal and COVID-19 viral pneumonia. Likewise, for the COVIDGR dataset, the proposed feature descriptor yielded better performance compared to most of the DL methods while achieving comparable performance with respect to method COVID-SDnet. Since the proposed approach is a machine learning model, it does not require specialized hardware, has less training time, obtains stabilized model with good detection performance with small training datasets, is lightweight, and can be deployed quickly. Future efforts will be focused on: (a) constructing a 3D feature descriptor that can help analyze 3D medical images, such as 3D CT images, by extracting the volume information and depth of spread of the disease, (b) detecting COVID-19 symptoms using multi-view based 3D shapes where the input data are taken from different angles, (c) testing surfaces for coronavirus detection, and (d) studying the long-term effects of COVID-19 from the patients recovered from the disease.

**TABLE V:** Comparative analysis of the proposed method with DL based methods used on the COVDIGR dataset.

			Non-COVID-19			COVID-19		
Author	Methods	Accuracy	F1-score	Precision	Specificity	F1-score	Precision	Recall
Tabik et. al [52]	COVDINet-CXR	$67.82\pm 6.11$	$73.31\pm 3.79$	$3.36\pm 6.15$	$88.82\pm 0.90$	$56.94\pm 15.05$	$81.65\pm 6.02$	$46.82\pm 17.59$
	COVID-CAPS	$65.34\pm 3.26$	$65.15\pm 5.02$	$65.62\pm 3.98$	$65.47\pm 9.93$	$64.87\pm 4.92$	$66.07\pm 4.49$	$64.93\pm 9.71$
	ResNet50 without segmentation	$74.25\pm 3.61$	$75.40\pm 4.91$	$71.91\pm 3.12$	$79.87\pm 8.91$	$72.69\pm 3.45$	$78.75\pm 6.31$	$68.63\pm 6.08$
	ResNet50 with segmentation	$74.60\pm 2.93$	$75.46\pm 2.97$	$73.36\pm 4.66$	$78.41\pm 7.09$	$73.40\pm 4.01$	$77.17\pm 4.79$	$70.80\pm 8.26$
	FuCiTNet	$74.35\pm 3.34$	$75.8\pm 3.18$	$72.00\pm 4.48$	$80.79\pm 6.98$	$72.35\pm 4.76$	$78.48\pm 4.99$	$67.90\pm 8.58$
	COVID-SDNet	$76.18\pm 2.70$	$76.94\pm 2.82$	$74.74\pm 3.89$	$79.76\pm 6.19$	$75.71\pm 3.35$	$78.67\pm 4.70$	$72.59\pm 6.77$
Our work*	Structural pattern1	$74.41\pm 4.11$	$75.77\pm 3.15$	$74.01\pm 6.01$	$77.93\pm 3.38$	$73.73\pm 2.48$	$76.05\pm 2.35$	$71.86\pm 6.14$
	Structural pattern2	$75.11\pm 1.76$	$75.86\pm 2.11$	$74.75\pm 3.61$	$77.72\pm 8.06$	$74.03\pm 3.15$	$76.41\pm 7.38$	$72.65\pm 6.83$
	Structural pattern3	$74.88\pm 2.26$	$74.28\pm 4.74$	$75.68\pm 7.82$	$73.30\pm 5.06$	$74.34\pm 4.28$	$73.47\pm 3.74$	$75.69\pm 8.17$
